# Revision Roux-en-y hepaticojejunostomy for a post-cholecystectomy complex vasculobiliary injury with complete proper hepatic artery occlusion: A case report and literature review

**DOI:** 10.1016/j.ijscr.2019.03.032

**Published:** 2019-04-09

**Authors:** Gunjan S. Desai, Prasad Pande, Rajvilas Narkhede, Dattaprasanna R. Kulkarni

**Affiliations:** a Department of Gastrointestinal Surgery, Lilavati Hospital and Research Center, Mumbai, Maharashtra, 400050, India; b Department of Gastrointestinal Surgery, Dr. Balabhai Nanavati Super Speciality Hospital, Ville Parle (West), Mumbai, Maharashtra, India

**Keywords:** Complete hepatic arterial ischemia, Complex biliary injury, Revision hepaticojejunostomy, Case report

## Abstract

**Introduction:**

Complete proper hepatic arterial [PHA] occlusion due to accidental coil migration during embolization of cystic artery stump pseudoaneurysm resulting from a complex vasculobiliary injurie [CVBI] post laparoscopic cholecystectomy [LC] is an extremely rare complication with less than 15 cases reported. We present a case depicting our strategy to tackle this obstacle in management of CVBI and review the relevant literature.

**Presentation of case:**

A 35 year old lady presented on sixth postoperative day with an external biliary fistula following Roux-en-y hepaticojejunostomy [RYHJ] for biliary injury during LC. She developed a leaking cystic artery pseudoaneurysm, during angioembolisation of which, one coil accidentally migrated into left hepatic artery resulting in complete PHA occlusion. Fourteen months later, cholangiogram revealed a worsening RYHJ stricture despite repeated percutaneous balloon dilatations. Multiple collaterals had developed. Revision RYHJ was fashioned to the anterior wall of biliary confluence with an extension into left duct. Minimum hilar dissection ensured preservation of collateral supply to the biliary enteric anastomosis. Postoperative recovery was uneventful. The patient is doing well at 1 year follow up.

**Discussion:**

Definitive biliary enteric repair should be delayed till collateral circulation is established within the hilar plate, hepatoduodenal ligament and perihepatic/peribiliary collaterals to provide an adequate arterial blood supply to biliary confluence and extrahepatic portion of the bile duct.

**Conclusion:**

Assessment of hepatic arteries should be part of investigation of all complex biliary injuries. Delayed definitive biliary enteric repair ensures a well-perfused anastomosis. Minimum hilar dissection is the key to preserve biliary and hepatic neovasculature.

## Highlights

• Vascular assessment is important in all complex biliary injury cases.
• Perihepatic/peribiliary collaterals provide adequate blood supply to bile ducts.
• Balloon dilatation is helpful in biliary-enteric anastomotic strictures.
• Delayed biliary enteric repair is better in proper hepatic artery block cases.
• Minimum hilar dissection should be done during definitive repair.

## 1 Introduction

Complex vasculobiliary injuries [CVBI] constitute 12–61% of biliary injuries, most commonly following laparoscopic cholecystectomy [1]. They present a formidable challenge to the multidisciplinary team treating these patients. During management, complete right and left hepatic arterial occlusion due to accidental coil migration during embolization of a cystic artery stump pseudoaneurysm is an extremely rare complication [2,3]. We present a case depicting our strategy to tackle this obstacle in the management of CVBI. This work has been reported in line with the SCARE criteria [4].

## 2 Case

A 35 year old healthy lady presented to our department on sixth postoperative day [POD] with an external biliary fistula and intra-abdominal sepsis. She had undergone laparoscopic converted to open cholecystectomy for acute calculous cholecystitis. She had a biliary injury that was identified intra-operatively, managed by Roux-en-y hepaticojejunostomy[RYHJ]. The anastomosis leaked. An interno-external percutaneous transhepatic biliary drainage[PTBD] extending across the leak was performed at our hospital on POD 7 for both right and left hepatic ducts.

**Fig. 1:**
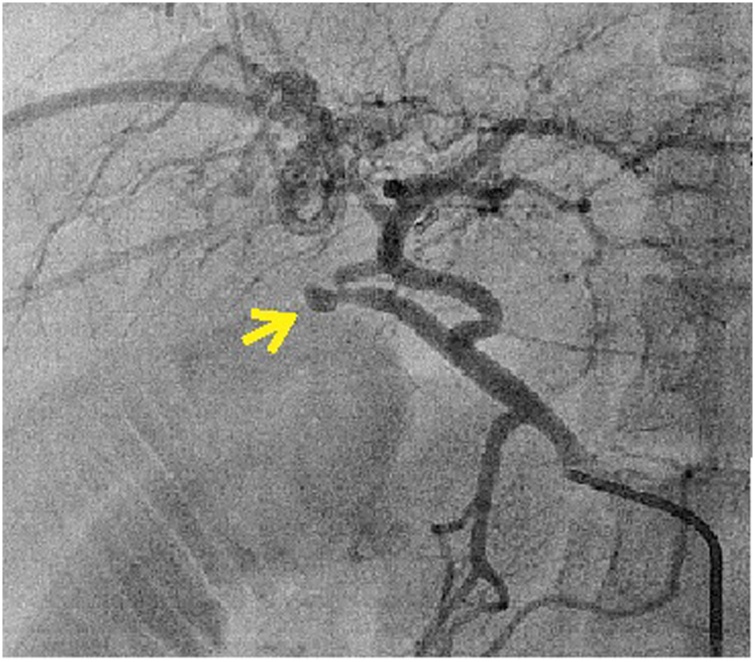
Conventional celiac artery angiogram showing the cystic artery pseudoaneurysm[yellow arrow].

**Fig. 2:**
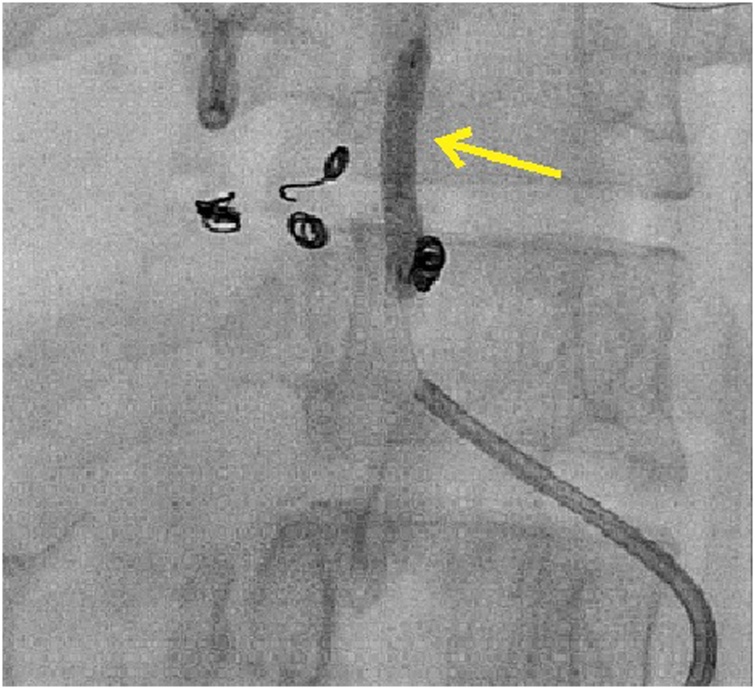
Conventional celiac artery angiography and coiling of cystic artery pseudoaneurysm and right hepatic artery and coil migration into left hepatic artery followed by stenting of the left hepatic artery[yellow arrow].

On POD nine, she had an upper gastrointestinal bleed. Esophagogastroduodenoscopy and Contrast enhanced computed tomography [CECT] abdomen did not reveal the source of bleeding. On conventional hepatic arteriogram, a leaking cystic artery pseudoaneurysm was identified (Fig. 1). During angioembolisation, due to a short stump of cystic artery, coils were placed in right hepatic artery [RHA]. However, one of the coils accidentally migrated into the left hepatic artery [LHA] and could not be retrieved. LHA stenting was performed, with good flow of contrast across the stent (Fig. 2). However, LHA developed spasm in its distal part, resulting in complete block of LHA and RHA.

On first day after coiling, there was significant elevation of liver enzymes with features of ischemic hepatitis. CECT abdomen with arteriography revealed poor enhancement of hepatic arterial tree in the segmental branches with partial revascularization from inferior phrenic and retroperitoneal arteries. The patient’s relatives were explained the possibility of a need for an emergency liver transplant. The patient improved over the next 48 h, was transferred out of intensive care unit and oral feeds were started. Abdominal drain was removed after it stopped draining bile. She was discharged on POD 28 with PTBD catheters in situ. On POD 33, her liver function tests were within normal limits, percutaneous transhepatic cholangiogram showed trickle of contrast across the RYHJ, and the PTBD catheters were clamped. PTBD catheters were kept longer than 6 months and intervening periodic cholangiograms revealed anastomotic narrowing. Balloon dilatation of the stenosed anastomosis was performed on multiple occasions. Liver function was normal all along.

**Fig. 3:**
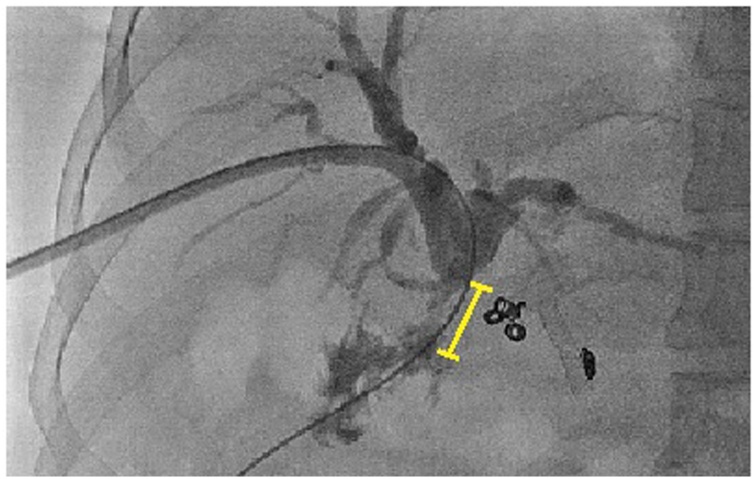
Percutaneous transhepatic cholangiogram showing the Roux-en-y hepaticojejunostomy anastomotic stricture.

**Fig. 4:**
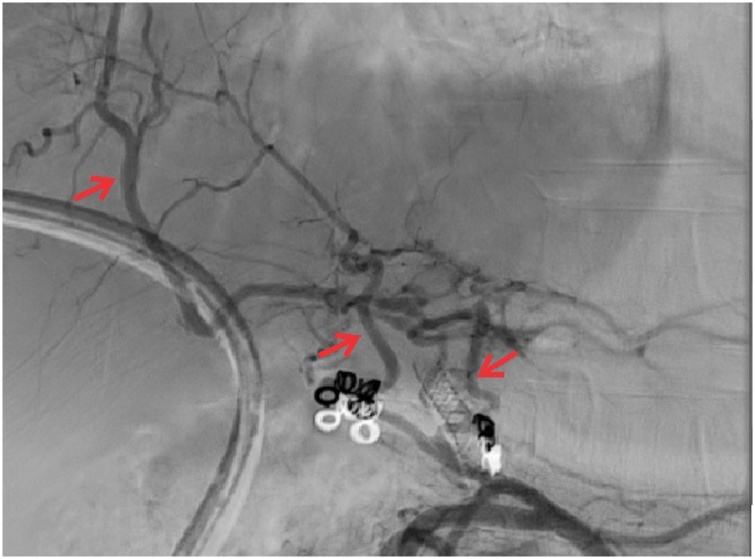
Conventional celiac artery angiogram showing collaterals[red arrows] from the proper hepatic artery, inferior phrenic artery and along the hepatoduodenal ligament.

Fourteen months after surgery, cholangiogram revealed a worsening of RYHJ stricture (Fig. 3). A CECT abdomen and conventional angiogram performed at 18 months showed a blocked RHA and LHA. Multiple collaterals were seen arising from the right inferior phrenic artery, retroperitoneum and along the hepatoduodenal ligament (Fig. 4). In view of the collateral formation and persistent tight stricture with failed multiple dilatations, a surgical revision of the anastomosis was planned. Prior to surgery, right PTBD catheter was maneuvered from right to left duct across the hilum, draining externally.

During surgery, utmost care was taken not to release any adhesions in the hepatoduodenal ligament and not to mobilize liver from the diaphragmatic attachments to preserve all collaterals. Hilar dissection was kept to a minimum and the PTBD catheters guided the identification of biliary hilar confluence. A minor segement IV hepatotomy was done to expose the left hepatic duct and the roof of biliary confluence. Previous anastomosis was resected, and a Roux loop was prepared. A 2 cm single layer, interrupted 5-0 polydioxanone RYHJ was fashioned to the anterior wall of the biliary confluence with an extension onto the left duct.

Her postoperative recovery was uneventful. Cholangiogram on POD 10 showed a good flow across the anastomosis with no sign of a leak, and she was discharged with a clamped PTBD catheter. The PTBD catheter was removed 6 weeks after the surgery. The patient is doing well at 1 year follow up. Liver function is normal. She is being followed up to evaluate for secondary patency.

## 3 Discussion

CVBI, most commonly seen after a laparoscopic cholecystectomy, is defined as any biliary injury that involves the confluence or extends beyond it, any biliary injury with major vascular injury or any biliary injury in association with portal hypertension or secondary biliary cirrhosis [5]. Major vascular injury is injury involving one or more of aorta, vena cava, iliac vessels, right hepatic artery, cystic artery, or the portal vein, seen in 0.04–0.18% of the operated patients and more so in patients with biliary injury [6]. The vascular injury is suspected intraoperatively when there is significant bleeding during laparoscopic cholecystectomy, when there is a sudden rise in alanine aminotransferase during early postoperative course, or when there are multiple metallic clips on plain film images of the abdomen [5]. The arterial injury may have been the result of the primary bile duct injury or may be the result of the attempted biliary repair [7,8]. Vascular injury can present as pseudoaneurysm, usually within the first 6 weeks of post-operative period, abscesses over 1–3 weeks or ischemic liver atrophy after many years [2,8].

Proper hepatic artery/both right and left hepatic artery involvement in CVBI has been very scarcely reported in literature. The reported cases in English literature and their management plan are presented in Table 1 [1,7,[9], [10], [11], [12], [13], [14]]. While these cases deal with intraoperative proper hepatic artery injury/occlusion, our case had a post RYHJ cystic artery pseudoaneurysm to begin with and the proper hepatic artery occlusion was an unfortunate event that took place during the embolization. There are occasional case reports of interventional proper hepatic artery pseudoaneurysm coiling leading to complete proper hepatic arterial occlusion with good outcome [15]. However, a biliary injury complicated by cystic artery pseudoaneurysm bleed and then a proper hepatic artery occlusion has not been reported so far, to the best of our knowledge.

**Table 1:** Reported cases of complex vasculobiliary injuries involving common hepatic artery or proper hepatic artery during cholecystectomy, their presentation, management and outcomes.

Sr no.	Year	Arterial injury	Biliary injury	Identification and Presentation	Management	Follow-up	Outcome
1	Frilling et al. [9]	Proper hepatic artery	E2	During first surgery	End-to-end repair of artery and Roux-en-Y hepaticojejunostomy – On POD 16, re-exploration showed ligated right hepatic artery and thrombosis of superior mesenteric vein and portal vein – right hepatectomy and listed for urgent liver transplantation	None	Death on POD 28 – could not undergo liver transplant
2	Frilling et al. [9]	Common hepatic artery	E1	POD 17	Reconstruction of artery using mesenteric venous graft and external drainage of both biliary ducts – Roux-en-Y hepaticojejunostomy after 3 days	None	Death on POD 37
3,4	Wudel et al. [10]	Common hepatic artery	Not available	Not available	Not available	Not available	Not available
5	Gupta et al. [11]	Proper hepatic artery	Strasberg E4	Postoperative day 4	Bilateral hepaticojejunostomies without vascular repair/reconstruction	One and half year +	Recovered with intermittent biliary strictures being managed with percutaneous transhepatic balloon dilatations
6	Buell et al. [7]	Common hepatic artery	Transected right hepatic duct	Average 8.5 days	Primary repair of artery and percutaneous external biliary drainage	None	Death
7	Buell et al. [7]	Common hepatic artery	Major biliary injury – Roux-en-Y hepaticojejunostomy in first surgery	Average 8.5 days	Right hepatectomy and posted for liver transplantation	None	Death
8	Buell et al. [7]	Common hepatic artery	Major biliary injury – Roux-en-Y hepaticojejunostomy in first surgery	Average 8.5 days	Orthotopic liver transplantation	Regular	Alive
9	Salman et al. [12]	Proper hepatic artery	E3	First surgery was open cholecystectomywith right hepatic artery ligation	Second surgery on postoperative day 3 – T-tube drainage and hemostasis	One year +	Recovered
					Percutaneous drainage of cholangitic liver abscess on POD 85		
					Third surgery on postoperative day 144 – Roux-en-Y hepaticojejunostomy		
10	Yan et al. [13]	Proper hepatic artery	E4	Roux-en-Y hepaticojejunostomy during first surgery{laparoscopic cholecystectomy} or first postoperative week of surgery without repair/reconstruction of artery	Secondary biliary cirrhosis and portal hypertension on follow up {Range of 5-148 months} – Underwent liver transplantation	Range of 4-17 months	Recovered
11	Strasberg et al. [14]	Proper hepatic artery	Necrosis of the intrahepatic biliary tree till fourth order branches	Postoperative day 6 with a large biliary fistula	Liver transplantation on Postoperative day 39	None	Death on post transplant day 14
12, 13	Sarno et al. [1]	Common hepatic artery	Major biliary injury	Details not available	Primary or revisional hepaticojejunostomy. Vascular repair/reconstruction not described	Follow up range of 12-245 months	Recovered

Proper hepatic arterial injury induces biliary ischaemia and worsens a biliary injury. The hepatic ischemia is usually transient. The effect is more profound when the biliary confluence is disrupted along with a proper hepatic artery injury, since the hilar marginal collateral is also disrupted in these injuries [8]. The effect on blood supply also affects the surgical outcomes. On univariate analysis in a study on factors affecting the surgical outcomes in biliary injury cases, VBI and sepsis were identified as factors for treatment failure. Also, 75% of these cases had complications and a poor long term patency rate [1]. 5.6–15% of CVBI cases in another published series required liver resection. Known indications of hepatectomy included concomitant vascular injury and high biliary injury, liver atrophy, and intrahepatic bile duct strictures in their series [2,8]. The clearing function of the liver with the translocated intestinal bacteria is impaired after ischemia and hence, it is important to maintain these patients with high antibiotic levels in the blood just to avoid septic complications in the ischemic liver parenchyma [5].

**Fig. 5:**
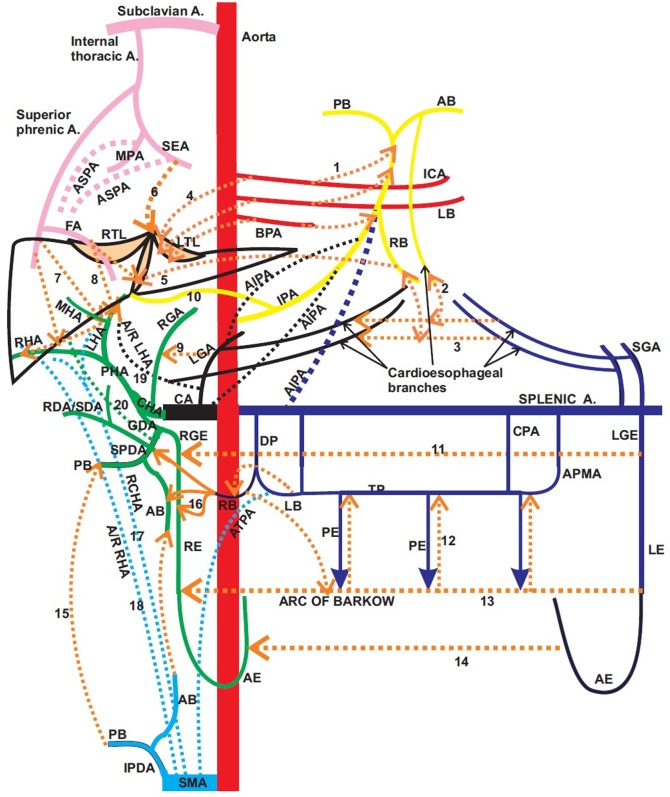
Schematic diagram to show the possible arterial collateral channels[numbered dotted lines] to the liver. SEA: Superior epigastric artery, MPA: Musculophrenic artery, ASPA: Accessory superior phreinc artery, ICA: Intercostal artery, AB: Anterior branch, PB: posterior branch, LB: Left branch, RB: Right branch, BPA: Branch to phrenic artery,IPA: Inferior phrenic artery, AIPA: Accessory inferior phrenic artery, RTL: Right triangular ligament, LTL: Left triangular ligament, FA: Falciform artery, RHA: Right hepatic artery, MHA: Middle hepatic artery, LHA: Left hepatic artery,CA: Celiac artery, LGA: Left gastric artery, CHA: Common hepatic artery, SPDA: Superior pancreaticoduodenal artery, PHA: Proper hepatic artery, GDA: Gastroduodenal artery, RDA/SDA: Retroduodenal artery/Supraduodenal artery, LGE: Left gastroepiploic artery, CPA: Caudal pancreatic artery, APMA: Arteriapancreatica magna, DP: Dorsal pancreatic artery, TP: Transverse pancreatic artery, AE: Anterior epiploic artery, PE: Posterior epiploic artery, RGE: Right gastroepiploic artery, A/R LHA: Accessory/Replaced left hepatic artery, A/R RHA: Accessory/Replaced right hepatic artery, RCHA: Replaced common hepatic artery, ATPA: Accessory transverse pancreatic artery, SMA: Superior mesenteric artery, IPDA: Inferior pancreaticoduodenal artery, LE: Left epiploic artery, RE: Right epiploic artery.

Table 1 shows that these cases have a very high mortality rate and overall outcomes are poor. The management options include hepatic resection, liver transplantation in cases with fulminant liver failure and revision RYHJ with or without an arterial repair. Most of these cases have been managed by an early attempted RYHJ and arterial repair [1,7,[9], [10], [11], [12], [13], [14]]. However, temporary percutaneous biliary intervention to allow the collaterals to form has not been attempted in these cases. Since Michel’s study on collateral circulation of liver to the present angiographic studies, it is now known that there are a lot of possible collateral channels to liver and bile ducts as shown in Fig. 5. Collateral flow can be demonstrated 10 h after the occlusion. With time, the collaterals are good enough to sustain the biliary system [16].

As seen in Fig. 5, collaterals can develop from common hepatic artery, gastroduodenal artery, ligamentous arteries from falciform, coronary and right triangular ligaments, pancreatoduodenal arteries, the intercostal arteries and the phrenic arteries [14,16]. In our case, the predominant collaterals were in the hepatoduodenal ligament and from superior and inferior phrenic arteries. Whilst waiting for the collateral supply to develop, the biliary anastomotic leak can be managed percutaneously by balloon dilatation and/or stenting as was performed in our case.

Surgical planning involves identification of all collateral channels on the arteriogram. During surgery, liver mobilization has to be kept to minimum to preserve the ligamentous collaterals and hepatoduodenal ligament dissection also needs to be minimum [5]. The revision anastomosis is performed with the standard surgical principles of RYHJ.

## 4 Conclusion

Vascular assessment should be part of investigation of all complex biliary injury cases. Delayed definitive repair in cases involving PHA occlusion to allow collateral circulation to be established within the hilar plate, hepatoduodenal ligament and perihepatic/peribiliary collaterals and thereby provide an adequate arterial blood supply to the biliary confluence and the extrahepatic portion of bile duct is a feasible management option. Minimum dissection should be done during surgery to preserve biliary and hepatic neovasculature.

## Conflicts of interest

Authors declare that they have no conflict of interest.

## Sources of funding

This research did not receive any specific grant from funding agencies in the public, commercial, or not-for-profit sectors.

## Ethical approval

Institutional ethics committee approval was taken for the publication.

## Consent

Informed consent was obtained from the patient for this publication.

## Author’s contribution

Gunjan Desai: collecting data, analysis of data, preparing the initial draft of the manuscript, critical revision of the manuscript for intellectual content, technical support, material support, study supervision.

Prasad Pande: critical revision of the manuscript for intellectual content, technical support, material support, study supervision.

Rajvilas Narkhede: critical revision of the manuscript for intellectual content, technical support, material support, study supervision.

Dattaprasanna Kulkarni: study concept, critical revision of the manuscript for intellectual content, administrative, technical support, material support, study supervision.

## Registration of research studies

This is a case report and does not require such registration. The publication was performed after the approval of research protocols by the Ethics Committee of Lilavati Hospital and Research Centre in accordance with international agreements (World Medical Association Declaration of Helsinki “Ethical Principles for Medical Research Involving Human Subjects,” amended in October 2013, www.wma.net).

## Guarantor

Gunjan S Desai is the article guarantor.

## Provenance and peer review

Not commissioned, externally peer-reviewed.
